# Exposure to the mycotoxin deoxynivalenol reduces the transport of conjugated bile acids by intestinal Caco-2 cells

**DOI:** 10.1007/s00204-022-03256-8

**Published:** 2022-02-28

**Authors:** Jingxuan Wang, Wouter Bakker, Weijia Zheng, Laura de Haan, Ivonne M. C. M. Rietjens, Hans Bouwmeester

**Affiliations:** Division of Toxicology, Wageningen University and Research, Stippeneng 4, 6708 WE Wageningen, The Netherlands

**Keywords:** Deoxynivalenol, Bile acid reabsorption, Conjugated bile acids, Bile acid transporters

## Abstract

Conjugated bile acids are synthesized in liver and subsequently secreted into the intestinal lumen from which they are actively reabsorbed and transported back to liver. The efficient enterohepatic circulation of conjugated bile acids is important to maintain homeostasis. The mycotoxin deoxynivalenol (DON) is a fungal secondary metabolite that contaminates cereal food. Upon human exposure, it can cause intestinal dysfunction. We explored the effects of DON exposure on the intestinal absorption of conjugated bile acids and the expression of bile acid transporters using an in vitro model based on Caco-2 cell layers grown in transwells. Our study shows that the transport rate of taurocholic acid (TCA) is decreased after 48-h pre-exposure of the Caco-2 cells to 2 µM DON, which is a realistic intestinal DON concentration. Exposure to DON downregulates expression of the genes coding for the apical sodium-dependent bile acid transporter (ASBT), the ileal bile acid-binding protein (IBABP) and the organic solute transporter α (OSTα), and it counteracts the agonist activity of Farnesoid X receptor (FXR) agonist GW4064 on these genes. In addition, the transport of ten taurine or glycine-conjugated bile acids in a physiological relevant mixture by the intestinal Caco-2 cell layers was decreased after pre-exposure of the cells to DON, pointing at a potential for DON-mediated accumulation of the conjugated bile acids at the intestinal luminal side. Together the results reveal that DON inhibits intestinal bile acid reabsorption by reducing the expression of bile acid transporters thereby affecting bile acid intestinal kinetics, leading to bile acid malabsorption in the intestine. Our study provides new insights into the hazards of DON exposure.

**Supplementary Information:**

The online version contains supplementary material available at 10.1007/s00204-022-03256-8.

## Introduction

Mycotoxins are toxic secondary metabolites produced by fungi that can contaminate cereal food commodities. Mycotoxin-contaminated food products pose a severe foodborne risk to human health worldwide. According to a study performed by the United Nations Food and Agriculture Organization (FAO), approximately 25% of the global food and feed that is produced annually is contaminated with mycotoxins (Boutrif and Canet 1998), which was confirmed more recently (Eskola et al. 2020). Among the various mycotoxins, the trichothecene toxin deoxynivalenol (DON) is one of the most prevalent and hazardous ones (Mishra et al. 2020). Because of the chemical stability, DON does not degrade during food processing and, therefore, can end up in the finished food product as consumed (Maresca 2013; Pinton and Oswald 2014). DON is absorbed through the intestinal epithelium, mainly by paracellular diffusion and is known to cause a variety of effects in the body (EFSA 2017; Sergent et al. 2006). Animal studies have revealed that chronic exposure to low concentration of DON alters intestinal functions resulting in malnutrition, diarrhea, vomiting and intestinal inflammation (Liu et al. 2020; Vignal et al. 2018). Intestinal in vitro studies have revealed that DON reduces the intestinal epithelial barrier function by reducing the expression of tight junction proteins and apoptosis of enterocytes (Lucioli et al. 2013; Pinton et al. 2009). Emerging evidence suggests DON is able to reduce the downstream gene transcription of the FXR/RXR pathway in porcine jejuna explants based on a pan-genomic transcriptomic analysis (Pierron et al. 2016). The Farnesoid X Receptor (FXR) pathway has an important regulatory function in intestinal bile acid metabolism (Jia et al. 2018). This suggests that a DON-contaminated diet may be an important risk factor interfering with intestinal bile acid homeostasis.

Bile acid homeostasis plays an important role in intestinal homeostasis. Primary bile acids are synthesized by hepatocytes and after conjugation with glycine (glyco-, G) or taurine (tauro-, T) secreted into bile. They subsequently are transported to the intestinal lumen where they are deconjugated and transformed into secondary bile acids by the intestinal microbiome (Jia et al. 2018). Thus, both conjugated and unconjugated bile acids can be found in the intestinal lumen, where most of the bile acids are taken up by intestinal epithelial cells and subsequently transported back to the liver. This enterohepatic circulation of bile acids is an extremely efficient process, with more than 90% of the intestinal bile acids being reabsorbed (Fromm and Kim 2010). Unconjugated bile acids can be reabsorbed by passive or facilitated absorption throughout all the small intestine segments, whereas conjugated bile acid is reabsorbed by active transport predominantly in the ileum. This active transport depends on the functioning of various bile acid transporters (Dawson et al. 2009). The apical sodium-dependent bile acid transporter (ASBT), ileal bile acid-binding protein (IBABP) and bile acid efflux transporters (i.e., the organic solute transporter (OST)) are the most important transporters (Dawson et al. 2009). These bile acid transporters can be regulated by multiple chemicals thus affecting the bile acid intestinal kinetics. It has been reported that these bile acid transporters are target genes of FXR, and regulated by FXR activity (Jia et al. 2018). Considering the effect of DON on FXR activity, we hypothesize that DON has the potential to induce bile acid malabsorption in the intestine by downregulating bile acid transporters.

The purpose of this study was to explore whether DON could reduce the intestinal reabsorption of bile acids and to study its potential mode of action using an in vitro testing strategy. To test our hypothesis, Caco-2 cell layers in transwells were pre-exposed to DON followed by exposure to bile acids to study their transport. Bile acid transport was quantified using an LC–MS/MS method, and intestinal barrier integrity was assessed by TEER measurements and Fluorescein paracellular transport marker evaluations. In addition, to investigate the mechanism underlying potential effects on the bile acid reabsorption, the mRNA expression of bile acid transporters was quantified following exposure of the cells to DON and/or FXR signaling pathway agonists.

## Materials and methods

### Cell culture

Human colon carcinoma Caco-2 cells (passage number 15–35) were grown at 37 °C with 5% CO_2_ in Dulbecco Modified Eagle’s Medium (DMEM) GlutaMax™ (4.5 g/L d-glucose and pyruvate; Gibco BRL, Breda, The Netherlands), supplemented with 10% heat-inactivated fetal calf serum, 1% non-essential amino acid and 1% penicillin/streptomycin (Gibco BRL). Cells were subcultured at 60–70% confluence using 0.05% trypsin (Gibco BRL).

### Cell viability

Caco-2 cells were seeded at 8 × 10^3^ cells/well in 96-well plate maintained in culture for 21 days, and the culture medium was used and changed every other day. Cytotoxicity of DON on the 21-day cultured Caco-2 cell layers was evaluated by the WST-1 assay. Briefly, 21-day cultured Caco-2 cells were exposed to 0–10 μM DON for 48 h, and subsequently, incubated with WST-1 reagents 2-(2-methoxy-4-nitrophenyl)-3-(4-nitrophenyl)-5-(2,4-disulfophenyl)-2H-tetrazolium) (Sigma-Aldrich, St. Louis, MO, USA). For this, WST was added at 10% of the medium volume and the absorbance was measured at 440 nm and 620 nm with a SpectraMax M2 microplate reader (Molecular devices, San Jose, CA, USA) up to 60 min. Data were obtained by subtracting the 620 nm signal from the 440 nm signal. The cell viability was expressed as percentage of the control group.

### In vitro intestinal Caco-2 cell layer barrier integrity

Caco-2 cells were seeded at 4 × 10^4^ cells/insert in 12-well polycarbonate membrane inserts with 0.4 μm pore size (Corning Costar, Schnelldorf, Germany) and maintained in culture for 21 days. The integrity of Caco-2 cell layers grown for 21 days was assessed using transepithelial electrical resistance (TEER) and Fluorescein transport measurements. Caco-2 cell layers were exposed to 0–10 μM DON for 48 h. The TEER was measured with Millicell^®^ ERS-2 Epithelial Volt-Ohm Meter (Millipore, Amsterdam, The Netherlands). TEER values were expressed as kΩ × cm^2^. For fluorescein measurement, the exposure medium was removed by rinsing the cell layers with Hank’s balanced salt solution (Gibco BRL) supplemented with 10 mM HEPES (transport medium). After 30 min of incubation in transport medium, 25 nmol Fluorescein (Sigma-Aldrich) in 0.5 mL transport medium was added to the apical cell compartment. After 1 h of incubation, the amount of fluorescence was measured in the basal compartment with SpectraMax M2 microplate reader. The excitation and emission wavelengths were 490 and 520 nm, respectively. The data are presented as the percentage of control group.

### Taurocholic acid (TCA) transport assay

Caco-2 cell layers grown in transwells for 21 days were exposed to 2 μM DON for 48 h. Then, the exposure medium was removed and the cell layers were gently rinsed with transport medium. After 30 min incubation in transport medium, 2.5 nmol TCA (Sigma-Aldrich) in 0.5 mL transport medium was added to the apical compartment. After 0–180 min incubation, the amount of TCA was measured in the basal compartment using LC/MS/MS.

### Mixed conjugated bile acids’ transport assay

Caco-2 cell layers grown in transwells for 21 days were exposed to 2 μM DON for 48 h. Then, the exposure medium was removed and the cell layers were gently rinsed with transport medium. After 30 min incubation in transport medium, 25 nmol mixed conjugated bile acids in 0.5 mL transport medium was added to the apical compartment. In the bile acid mixture, conjugated bile acids were present in the same ratio as in human serum (Bathena et al. 2013). In the mixture GCDCA, GCA, GDCA, TCDCA, TCA, GUDCA, TDCA, GLCA, TUDCA and TLCA were present in the following concentrations: 16.5, 9.5, 8.0, 6.5, 3.5, 3, 2.5, 0.3, 0.2 and 0.05 μM, respectively. After 0–180 min incubation, the amount of each conjugated bile acid was measured in the basal compartment using LC/MS/MS.

### Profiling of bile acids by LC/MS/MS

Chromatographic separation of bile acids was carried out using the Shimadzu 8045 System (Kyoto, Japan). Aliquots of samples (1 μL) were injected by autosampler, and the analytes were separated on Phenomenex AJ0-8782 as a guard column and Phenomenex 00B-4475-AN (50 mm × 2.1 mm × 1.7 μm × 100 Å, Kinetex C18) was used as an analytical column at a column temperature of 50 °C. The mobile phase consisted of MilliQ water with 0.01% formic acid (A) and methanol with 50% acetonitrile (B) using a starting gradient 30% B, 70% B at 7.5 min, 98% B at 7.6–10.0 min, and then immediate reduction to 30% B at 10.5–16 min with re-equilibration before the next injection. The flow rate was 0.65 mL/min. The optimal ESI source parameters were as follows: nebulizer gas, 3 L/min; heating gas and drying gas, 10 L/min; interface temperature, desolvation line (DL) temperature and heat block temperature were maintained 300 ℃, 250 ℃ and 400 ℃, respectively. The multiple reaction monitoring (MRM) and selective ion monitoring (SIM) modes were employed for quantification. Data were collected and processed using the LabSolutions software (Shimadzu). The chromatogram of bile acid profiling and quantification table is provided in Supplementary materials Fig. S1 and Table S1.

### mRNA extraction and RT-qPCR analysis

21-day cultured Caco-2 cell layers were exposed to 2 μM DON or 2 μM synthetic FXR ligand GW4064 (Sigma-Aldrich) for 48 h depending on the experiment. RNA was isolated using the QIAshredder and RNeasy^®^ mini kit (Qiagen, Hilden, Germany) according to the manufacturer’s instructions. The quantity and quality of isolated mRNA were confirmed by Nanodrop (ND‐1000; ThermoScientific, Waltham, MA, USA). Subsequently, cDNA was generated from 300 ng of total RNA using the QuantiTect^®^ reverse transcription kit (Qiagen). RT-qPCR analysis was carried out using the Rotor‐Gene^®^ SYBR^®^ Green PCR kit and the Rotor‐Gene^®^ 6000 cycler according to the manufacture’s handbook. The human-specific primers we used were commercially available QuantiTect^®^ primer assays, including OSTα, IBABP, ASBT and β-actin. The FXR primer (Biolegio, Nijmegen, The Netherlands) was synthesized according to the sequence (F:-CTACCAGGATTTCAGACTTTGGAC, R:GAACATAGCTTCAACCGCAGAC). The efficiency of the primers was checked prior to sample measurement. Values were quantified using the comparative threshold cycle method and target gene mRNA expression was normalized to β-actin (Pfaffl et al. 2001). The data are presented as percentage of control group.

### Statistics

Data are presented as the means of three biological replicates ± SD. Statistical analysis was performed by Student *t* test or ANOVA followed by the Duncan test. *p* < 0.05 was considered as statistically significant. All data were analyzed with SPSS Version 17.0 (SPSS Inc., Chicago, IL, USA).

## Results

### DON reduces the integrity of Caco-2 cell layer

**Fig. 1:**
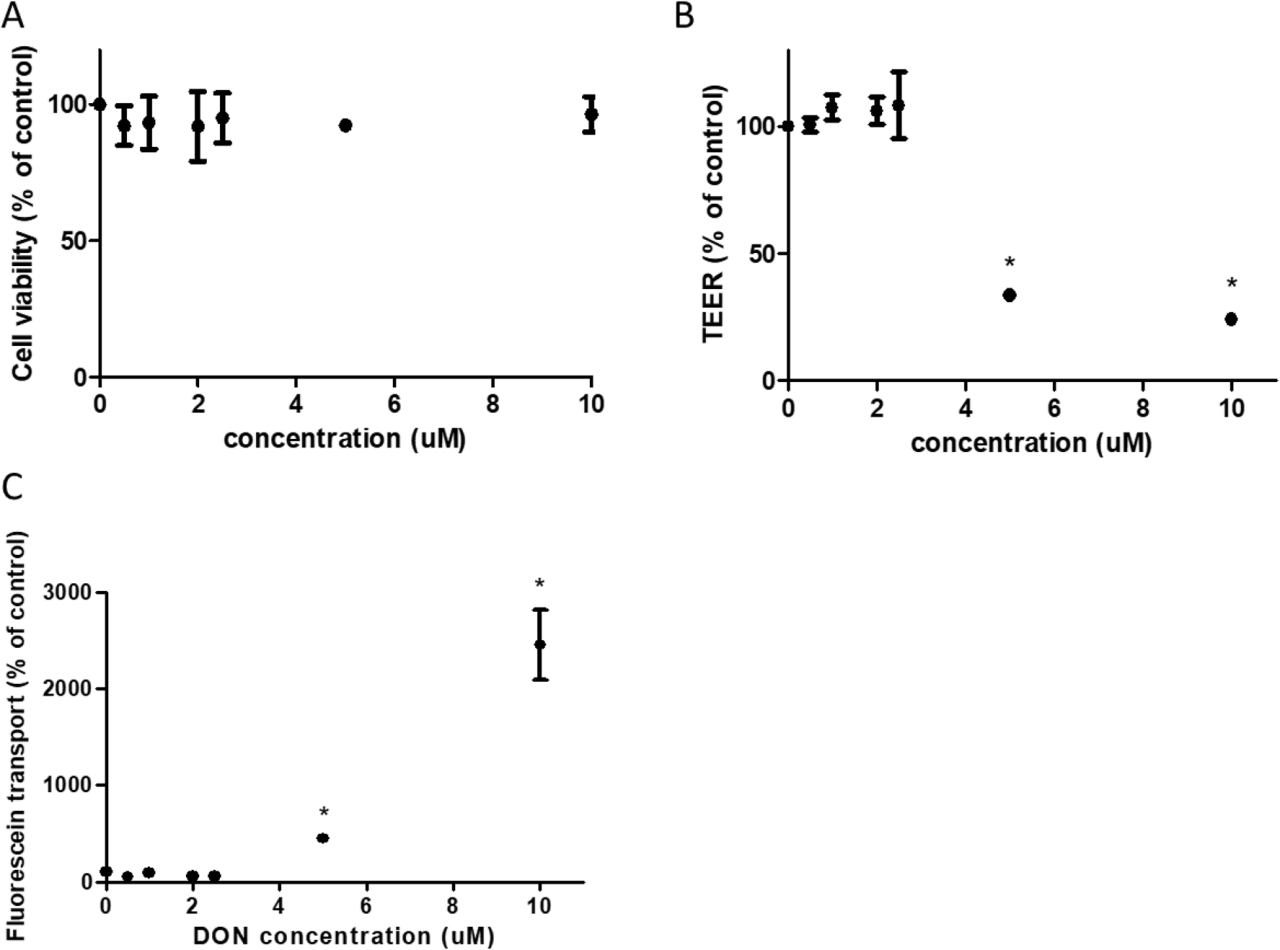
DON reduces the integrity of 21-day cultured Caco-2 cell layers. The viability of Caco-2 cells following exposure to different concentrations of DON (0–10 µM, 48 h) was analyzed by WST-1 assay (**A**). The effect of DON on the integrity of Caco-2 cell layer was assayed by the TEER measurement (**B**) and Fluorescein transport (**C**). Data were expressed as mean ± SD, *n* = 3. *Significantly different from the control group (*p* < 0.05)

For our studies, we needed to select a concentration of DON that does not affect the viability of 21-day cultured Caco-2 cell layers (viability of pre-confluent Caco-2 cells exposed to DON is shown in supplementary Fig. S2). We, therefore, exposed 21-day cultured Caco-2 cell layers to increasing concentrations of DON (0–10 µM) for 48 h. Mitochondrial activity in the 21-day cultured Caco-2 cells, as measured by the WST-1 assay, was not affected by the DON concentrations tested (Fig. 1A). In addition, we assessed whether exposure to DON disrupted the barrier properties of 21-day cultured Caco-2 cell layers. For this, we assessed the effect of DON on TEER and paracellular fluorescein translocation. Only DON concentrations of 5 and 10 µM affected the integrity of the Caco-2 cell layers as can be seen from the reduction of TEER values (less than 33.54 ± 0.35%) and the increase in fluorescein permeation (more than 450.92 ± 15.73%) compared to control group (Fig. 1B and C). Exposure of Caco-2 cell layers to 2.5 μM DON and lower concentrations did neither alter the cell layer barrier properties nor did it reduce the cell viability. Based on these results, we selected a DON concentration of 2 µM for the subsequent studies.

### DON decreases the TCA active transport across a Caco-2 cell layer

**Fig. 2:**
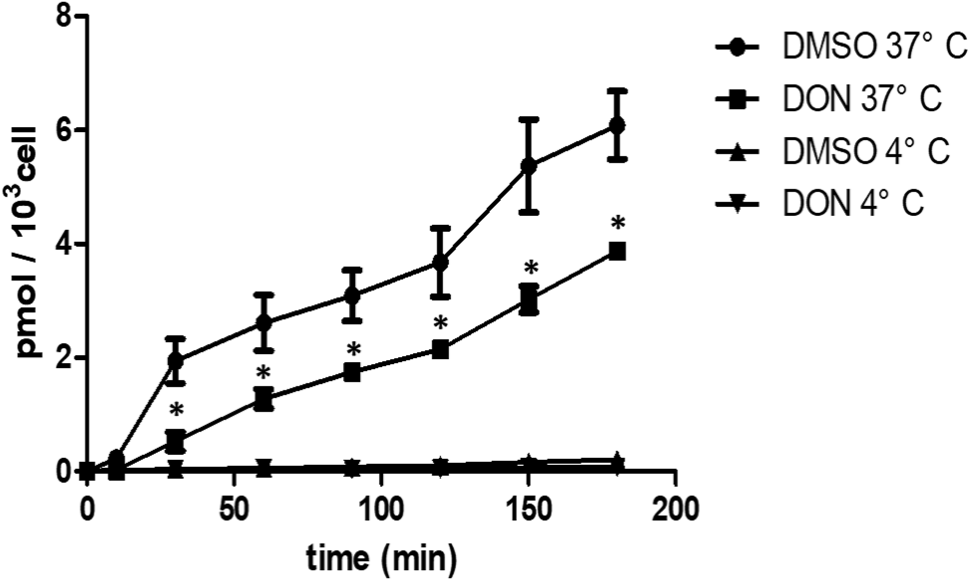
Transport of TCA across a Caco-2 cell layer measured during 180 min at 37 °C or 4 °C with or without DON pre-exposure. 21-day cultured Caco-2 cell layers were incubated with or without DON (2 µM, 48 h) before exposure to 2.5 nmol TCA in 0.5 mL apical transport medium. TCA transport from the apical to basolateral compartment was determined during 180 min at 37 °C or 4 °C. Data are expressed as mean ± SD, *n* = 3. *Mean values differ significantly from the DMSO control group at same time point (*p* < 0.05)

As tauro-conjugated bile acid, TCA is reabsorbed by intestinal bile acid transporters in the terminal ileum (Dawson and Karpen 2015). To assess the effect of DON on TCA active transport across Caco-2 cell layers, 21-day cultured cell layers were pre-incubated with 2 µM DON for 48 h. This concentration of DON was shown to be non-toxic. Following DON pre-exposure, the Caco-2 cell layers were incubated with 2.5 nmol TCA in 0.5 mL transport medium in the apical compartment for 180 min. TCA could not be detected in the basal compartment during 180 min transport when the transport experiment was performed at 4 °C (with or without DON pre-incubation). However, at 37 °C, TCA transport was significantly reduced following DON pre-exposure, the rates of TCA transport were 0.021 and 0.032 pmol/10^3^ cells per min with or without DON pre-exposure, respectively (Fig. 2).

### DON downregulates the mRNA expression of bile acid transporters in Caco-2 cell layers

**Fig. 3:**
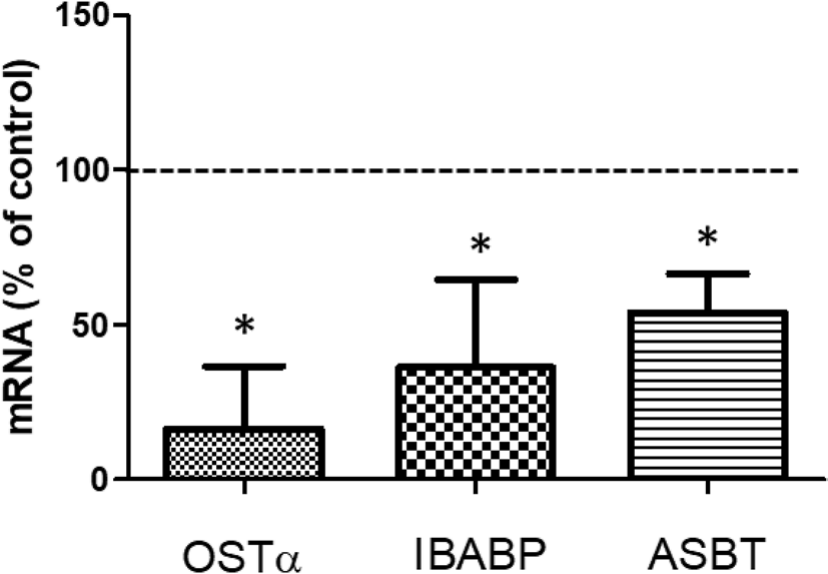
DON downregulates the mRNA expression of bile acid transporters in Caco-2 cell layers. The mRNA expression of OSTα, IBABP and ASBT in Caco-2 cell layers after exposure 2 µM DON for 48 h was analyzed by RT-qPCR and compared to 0.5% DMSO which was set at 100%. Data were expressed as mean ± SD, *n* = 3. *Significantly different from the control group (*p* < 0.05)

TCA can be transported from the intestinal lumen into the enterocyte by the apical sodium-dependent bile acid transporter (ASBT). In the cytoplasm of the enterocyte, TCA binds to the ileal bile acid-binding protein (IBABP) and is subsequently presented to the organic solute transporter (OST) that is present in the basal membrane via which it will be excreted (Meier and Stieger 2002). To explore the mechanism of the DON-induced decrease of TCA transport across the Caco-2 cell layers, the mRNA expression of bile acid transporters was analyzed by RT-qPCR. As shown in Fig. 3, the mRNA expression of the OSTα, IBABP and ASBT was decreased to 16.28 ± 16.55%, 36.22 ± 23.14% and 53.99 ± 12.42% compared to the control group, respectively, after exposure to 2 µM DON for 48 h.

**Fig. 4:**
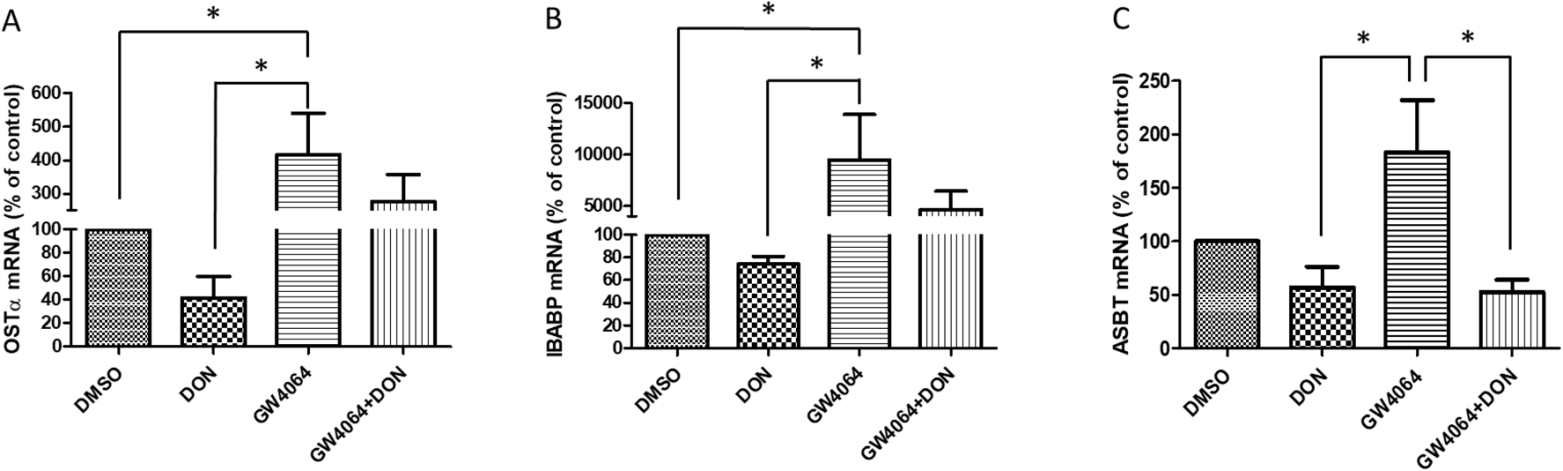
Effect of DON, GW4064 and combined DON + GW4064 exposure on the mRNA expression of bile acid transporters in Caco-2 cell layers. The mRNA expression of OSTα (**A**), IBABP (**B**) and ASBT (**C**) in 21-day cultured Caco-2 cell layers incubated with 50 µM GW4064 or co-exposed to 50 µM GW4064 and 2 µM DON for 48 h was analyzed by RT-qPCR. Data were expressed as mean ± SD, *n* = 3. *Significantly different between the indicated groups (*p* < 0.05)

The gene expression of bile acid transporters can be influenced by FXR, which is a member of the nuclear receptor superfamily of ligand-activated transcription factors (Jia et al. 2018; Wang et al. 2008). Exposure of the 21-day cultured Caco-2 cells to DON did not affect the FXR gene expression itself (Fig. S3). However, following exposure to the FXR agonist GW4064, the mRNA expression of OSTα and IBABP increased significantly, i.e., 4.17 ± 2.14 and 113.50 ± 77.37 times, respectively, compared with the control (Fig. 4A and B). For ASBT, the expression increased 1.83 ± 0.85 times compared with the control (N.S.) following exposure to the FXR agonist GW4064. This proves that OSTα and IBABP are downstream genes, regulated by FXR activity, while this cannot be concluded for ASBT since the effect of the FXR on the ASBT mRNA expression was not significant.

In line with the results presented in Fig. 3, exposure of the 21-day cultured Caco-2 cells to 2 µM DON decreased the mRNA expression of OSTα, IBABP and ASBT compared to the control. Exposure to DON also reduced the FXR agonist GW4064-mediated increase in the mRNA expression of the three transporters OSTα, IBABP and ASBT. The GW4064-mediated induction of OSTα and IBABP was no longer significant in the presence of DON while this DON-mediated reduction in GW4064-induced mRNA expression appeared significant especially in the case of ASBT mRNA (Fig. 4A–C).

### DON decreases the conjugated bile acid transport through Caco-2 cell layers

**Fig. 5:**
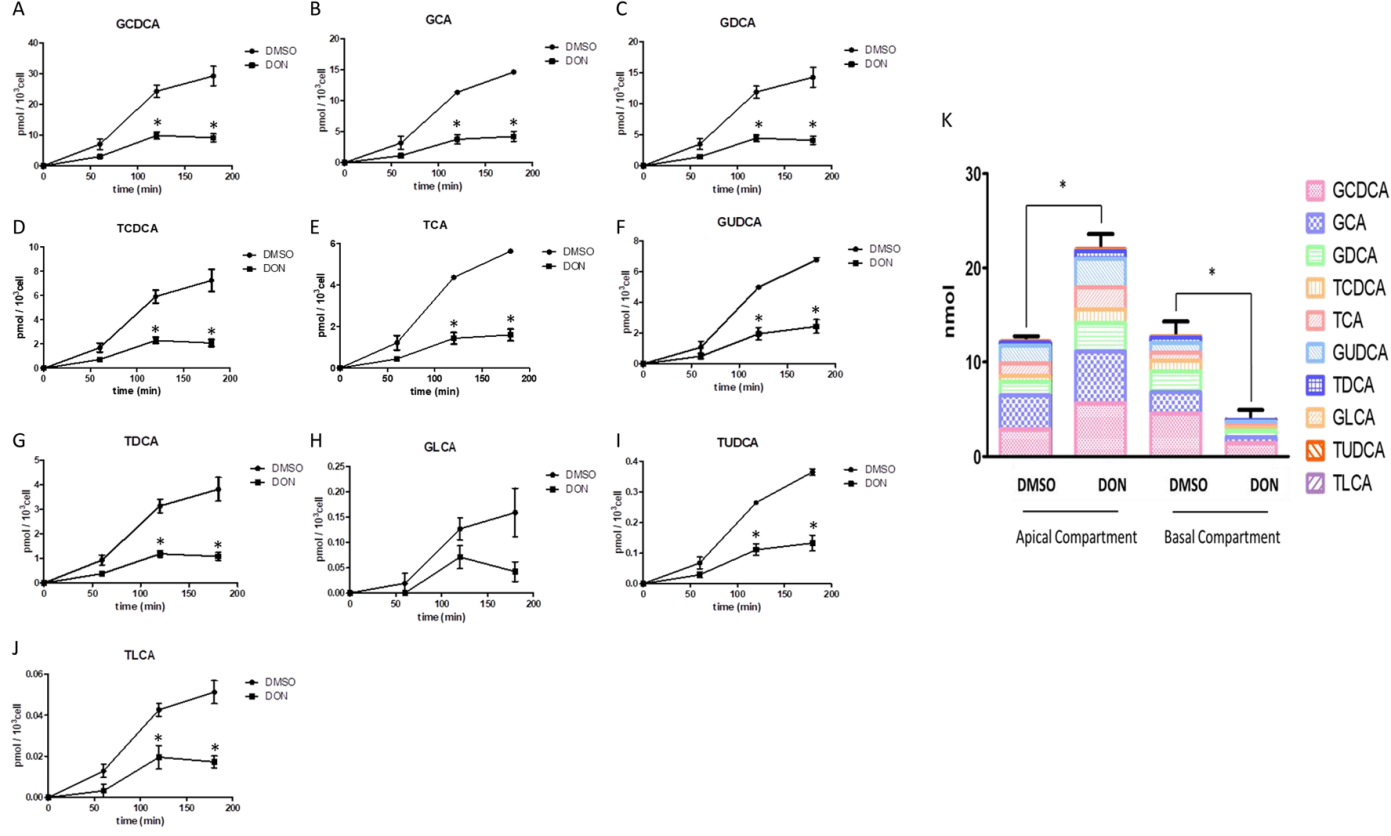
DON decreases the conjugated bile acid transport through Caco-2 cell layer. 21-day cultured Caco-2 cells were incubated with DMSO or DON (2 µM, 48 h) before exposure to 50 µM physiological mix of conjugated bile acids in 0.5 mL apical transport medium. The concentrations of GCDCA, GCA, GDCA, TCDCA, TCA, GUDCA, TDCA, GLCA, TUDCA and TLCA in the mixture is 16.5, 9.5, 8.0, 6.5, 3.5, 3, 2.5, 0.3, 0.2 and 0.05 μM, respectively. **A**–**J** The time course of each conjugated bile acid transport. **K** The amount of the different bile acids in the upper and basal compartment after 3 h transport. Data were expressed as mean ± SD, *n* = 3. *Mean values differ significantly between the control group at same time point (*p* < 0.05)

Finally, we not only assessed the transport of one single bile acid (see Fig. 2), but assessed if exposure to DON affects the transport of the individual conjugated bile acids if Caco-2 cells are exposed to 50 µM conjugated bile acids (i.e., a physiological relevant mixture, see “Materials and methods” section for composition) (Bathena et al. 2013). As shown in Fig. 5A–J, the transport of all these ten conjugated bile acids decreased after DON pre-exposure and the same trend can also be seen in the transport upon exposure of the Caco-2 cells to higher concentrations of the mixture of conjugated bile acids (Fig. S4A and B). After 3-h transport, the amount of total conjugated bile acids in the upper compartment was 12.21 ± 0.65 and 22.00 ± 2.16 nmol without or with DON pre-exposure, respectively. In addition, the amount of total conjugated bile acids in the basal compartment was 12.72 ± 1.60 and 3.85 ± 1.04 nmol without or with DON pre-exposure, respectively (Fig. 5K). The results indicated that the total conjugated bile acid transport through Caco-2 cell layer decreased significantly which results in a higher remaining concentration in the apical compartment after pre-exposure with the 2 µM DON. However, the relative proportion of each conjugated bile acid in the total bile acid after transport was not much different comparing the results with or without pre-exposure to DON. GCDCA and GCA were the most abundant ones remaining in the upper compartment, with amounts that increased from 2.85 ± 0.21 to 5.60 ± 1.13 nmol and 3.61 ± 0.28 to 5.50 ± 0.36 nmol after pre-exposure to DON.

## Discussion

In this study, we demonstrated that exposure to the mycotoxin deoxynivalenol (DON) reduced the active transport of conjugated bile acids across Caco-2 cell layers and decreased the expression of bile acid transporters OSTα, IBABP and ASBT in the cells. In addition, DON counteracted the agonist activity of Farnesoid X receptor (FXR) agonist GW4064 on these genes pointing at the involvement of the FXR pathways in this DON-mediated effect on bile acid transporters and transport.

Human exposure to DON has long been associated with intestinal dysfunction (Maresca and Fantini 2010). To protect the consumer from the adverse health effects of DON, the European Food Safety Authority (EFSA) established a TDI of 1 µg/kg bw based on a reduced body weight gain in mice (EFSA 2017). Based on this, the permissible level of DON in cereal flours for direct human consumption is 750 μg/kg in the European Union (EFSA 2017), and 1000 μg/kg in the United States (EFSA 2017; Mishra et al. 2020). We aimed to use a realistic DON concentration in our in vitro studies. Assuming that maximal tolerated DON levels are present and ingested in one meal, and that upon ingestion this is diluted in 1 L of gastrointestinal fluid and is totally bio-accessible, an intestinal lumen concentration of 2.5–3.3 μM DON can be estimated (EFSA 2017; Mishra et al. 2020).

Exposure of 21-day cultured Caco-2 cell layers to 2.5 μM DON and lower concentrations did neither alter the cell layer barrier properties nor did it reduce the cell viability, which was in agreement with previous studies on differentiated Caco-2 cell layers (Bony et al. 2006; Wang et al. 2019). However pre-exposure to 2 μM DON decreased the amount of TCA transported across the Caco-2 cell layers. TCA has long been used as a model bile acid for the study of bile acid active transport (Heubi et al. 1982; Li et al. 2018). In vivo, decreased bile acid active transport may lead to disturbance of bile acid homeostasis, as most of the bile acid pool in human intestine is conjugated to taurine or glycine metabolites. Their active uptake across the apical brush border membrane and the basal membrane requires the presence of ileal transporters (Northfield and McColl 1973). Therefore, we subsequently studied the expression and the regulation thereof of the bile acid transporters IBABP, OSTα and ASBT, which are the specific transporters for taurine or glycine-conjugated bile acids (Fromm and Kim 2010).

The ileal ASBT mediates the initial uptake of bile acids across the ileal enterocyte apical brush border membrane, only a small fraction of the taurine or glycine bile acid metabolites escapes absorption and is excreted in the feces (Li et al. 2018). In our study, the mRNA expression of ASBT was decreased following DON exposure. It has been reported previously that a downregulation of ASBT is related to the decrease of TCA influx into differentiated Caco-2 cells (Thomas et al. 2006). In addition, clinical research found that mutations of the ASBT gene are associated with primary bile acid malabsorption (Oelkers et al. 1997). After entering the cytosolic compartment, the bile acids bind to IBABP. The exact role of IBABP in the intestinal bile acid transport is still unclear. Some researchers reported that IBABP is involved in the intracellular bile acid transport (Kramer et al. 1993; Lin et al. 1990). In our study, the mRNA expression of IBABP was decreased following DON exposure, which suggested that the observed reduced TCA transport might be affected by the reduced IBABP mRNA expression. However, Kok et al. (2003) reported that mice lacking appreciable intestinal IBABP expression still exhibit normal intestinal bile acid absorption (Kok et al. 2003). Emerging in vitro evidence shows IBABP protects the enterocyte from the cytotoxic properties of bile acids (D’Onofrio et al. 2017). Decreased IBABP might suggest a risk of bile acid cytotoxicity in the cytoplasm following DON exposure potentially contributing to impaired integrity of the barrier function. Lastly, the basolateral membrane transporter OSTα/β is important for exporting conjugated bile acids from the enterocyte into the portal circulation. In our study, the mRNA expression of OSTα was decreased following DON exposure. It has been reported that a reduced ileal expression of OSTα/β led to low ileal bile acid reabsorption (Renner et al. 2008). After transferring the human OST gene into the MDCK cell, the uptake of TCA by the OST/MDCKII cell was significantly increased, which indicated the important role of OST in bile acid transport (Suga et al. 2019).

The expression of bile acid transporters is regulated via nuclear receptors. The FXR is a member of the superfamily of nuclear receptors and mainly expressed in the ileum and liver. Once activated, it binds to the FXR-responsive elements on the promoters of target genes and regulates the transcription of target genes involved in bile acid synthesis, transport and metabolism. Both IBABP and OST have been reported as target genes of FXR (Jia et al. 2018). In our study, the mRNA expression of OSTα and IBABP was increased following exposure to the FXR agonist GW4064 which proved that OSTα and IBABP are downstream genes, regulated by FXR activity. This corroborates earlier observations in which it has been reported that the FXR agonists GW4064 induced OST expression and the effect was blocked by FXR siRNA in Huh7 cells (Landrier et al. 2006). The GW4064-mediated induction of OSTα and IBABP was no longer significant in the presence of DON, indicating that DON could counteract the agonist activity of GW4064 on these genes. IBABP gene expression is transcriptionally activated by FXR through an FXR-responsive element in the IBABP promoter (Campana et al. 2005). It has also been reported that a decreased activity of FXR was associated with a reduction of IBABP expression in intestinal epithelial HT-29 cells in vitro (Gadaleta et al. 2011). The regulation of the ASBT expression by the FXR is controversial. In our study, for the gene expression of ASBT the effect of the FXR agonist was less strong as for OST and IBABP, with a non-significant increase up to 183 ± 85% of the control. This effect of the FXR agonist was fully prevented upon co-exposure with DON, since co-exposure to the FXR agonist and DON resulted in a similar reduction of ASBT expression as what was observed with DON alone. It has been reported that GW4064 increased the ASBT expression in ileum of rats and enhanced bile acid reabsorption, making it potential candidate for the treatment of bile acid malabsorption (Cao et al. 2019). Together, DON can not only cause bile acid malabsorption, but also counteracts the agonist activity of GW4064 on bile acid transporter genes, which increases the hazard of DON exposure on bile acid malabsorption.

Experiments with FXR-null mice suggested that the ASBT expression could also be downregulated through a pathway independent of FXR (Miyata et al. 2009). Alternative to the FXR pathway regulation of ASBT expression via the c-Fos pathway has been postulated. c-Fos is a critical mediator of the repression of ASBT expression (Neimark et al. 2006). c-Fos phosphorylation results in an activation and translocation from cytoplasm to the nucleus where it forms a heterodimer with c-Jun which binds to dAP-1. This dAP-1/protein complex prevents the bile acid transporter ASBT transcription (Chen et al. 2002). It has been reported that DON induced the DNA-binding activities of c-Fos/c-jun in Raw 264.7 macrophage cells (Wong et al. 2002). Taken together, it may be concluded that DON might also regulate the ASBT expression via c-Fos pathway. Besides, DON is known to target the ribosome and inhibits the chain elongation step of protein synthesis, leading to an inhibition of RNA, DNA and protein synthesis (EFSA 2017; de Loubresse et al. 2014; Pestka 2010). The direct induction of the ribotoxic stress by DON will not only affect the downregulation of ASBT, but also that of OSTα, IBABP. This remains an interesting topic for future research.

We lastly evaluated the consequence of a reduced expression of the bile acid transporters on the uptake and transport of a physiological relevant mixture of taurine and glycine-conjugated bile acids. The transport of conjugated bile acids by the intestinal Caco-2 cell layer was decreased after pre-exposure of the cells to DON, which resulted in a higher remaining concentration of conjugated bile acids in the upper compartment. If extrapolated to the in vivo situation, this would lead to increased levels of taurine and glycine-conjugated bile acids in the terminal ileum. The conjugated bile acids which are not reabsorbed at the terminal ileum will continue to move with the intestinal contents to reach the colon and undergo transformations such as deconjugated, oxidation and epimerization by gut bacteria (Jia et al. 2018). There is considerable evidence that bile acids can modify colonic motility, ultimately leading to diarrhea and dysfunction of absorption in the intestine (Bajor et al. 2010). In our study, after exposure to a realistic ratio of mixed conjugated bile acids, the relative proportion of each conjugated bile acid in the total bile acid after transport was not much different comparing the results with or without pre-exposure to DON. GCDCA and GCA are still the most abundant ones in the mixture remaining in the upper compartment following pre-exposure to DON, which can be deconjugated to CDCA and CA in the colon. CA and CDCA affected folding of different subsets of bacterial proteins, showing antibacterial function and reducing the diversity of gut microbiota (Dawson and Karpen 2015). Glycine, CA and TCA are well known to facilitate in vitro growth of *Clostridium difficile*, a pathogen responsible for significant morbidity of IBD and mortality in patients (Rupnik et al. 2009; Sorg and Sonenshein 2008). CA can be converted to DCA by bacterial dihydroxylation, which, combined with DCA formation via deconjugating of GDCA and TDCA, may result in a bile acid pool with more DCA that is more hydrophobic and toxic (Ridlon et al. 2006). It has been reported that excessive DCA in the intestinal lumen increased rabbit intestinal permeability (Fasano et al. 1990). The increase of intestinal permeability is a characteristic of intestinal dysfunction, including inflammation intestinal disease where it contributes to propagation and exacerbation of inflammation.

## Conclusion

Our study shows that DON decreases the active transport of conjugated bile acids in a Caco-2 cell layer. DON downregulates the expression of the bile acid transporters ASBT, IBABP and OSTα, and counteracts the agonist activity of the FXR agonist GW4064 on these genes. The decrease of the bile acid transporters can be expected to induce the malabsorption of conjugated bile acids in the intestine. Overall, it is concluded that DON affects the expression of the bile acid transporters and thereby bile acid intestinal kinetics, which provides new insights into the hazards of DON exposure.

## Supplementary Information

Below is the link to the electronic supplementary material.

Supplementary file1 (DOCX 449 KB)
