# Supplementary material for: Exposure to the mycotoxin deoxynivalenol reduces the transport of conjugated bile acids by intestinal Caco-2 cells
Source: Arch Toxicol. 2022 Feb 28;96(5):1473–82. doi: 10.1007/s00204-022-03256-8 (PMC9013688; doi:10.1007/s00204-022-03256-8)
Supplement: Supplementary file 1 — Supplementary file1 (DOCX 449 KB) [file 204_2022_3256_MOESM1_ESM.docx]

**Exposure to the mycotoxin deoxynivalenol reduces the transport of conjugated bile acids by intestinal Caco-2 cells**

**Jingxuan Wang^1^*, Wouter Bakker^1^, Weijia Zheng^1^, Laura de Haan^1^, Ivonne M.C.M. Rietjens^1^, Hans Bouwmeester^1^**

**^1^**Division of Toxicology, Wageningen University and Research, Stippeneng 4, 6708 WE Wageningen, The Netherlands

*Corresponding author: Jingxuan Wang: [Jingxuan.wang@wur.nl](mailto:Jingxuan.wang@wur.nl)

**Supplementary materials:**


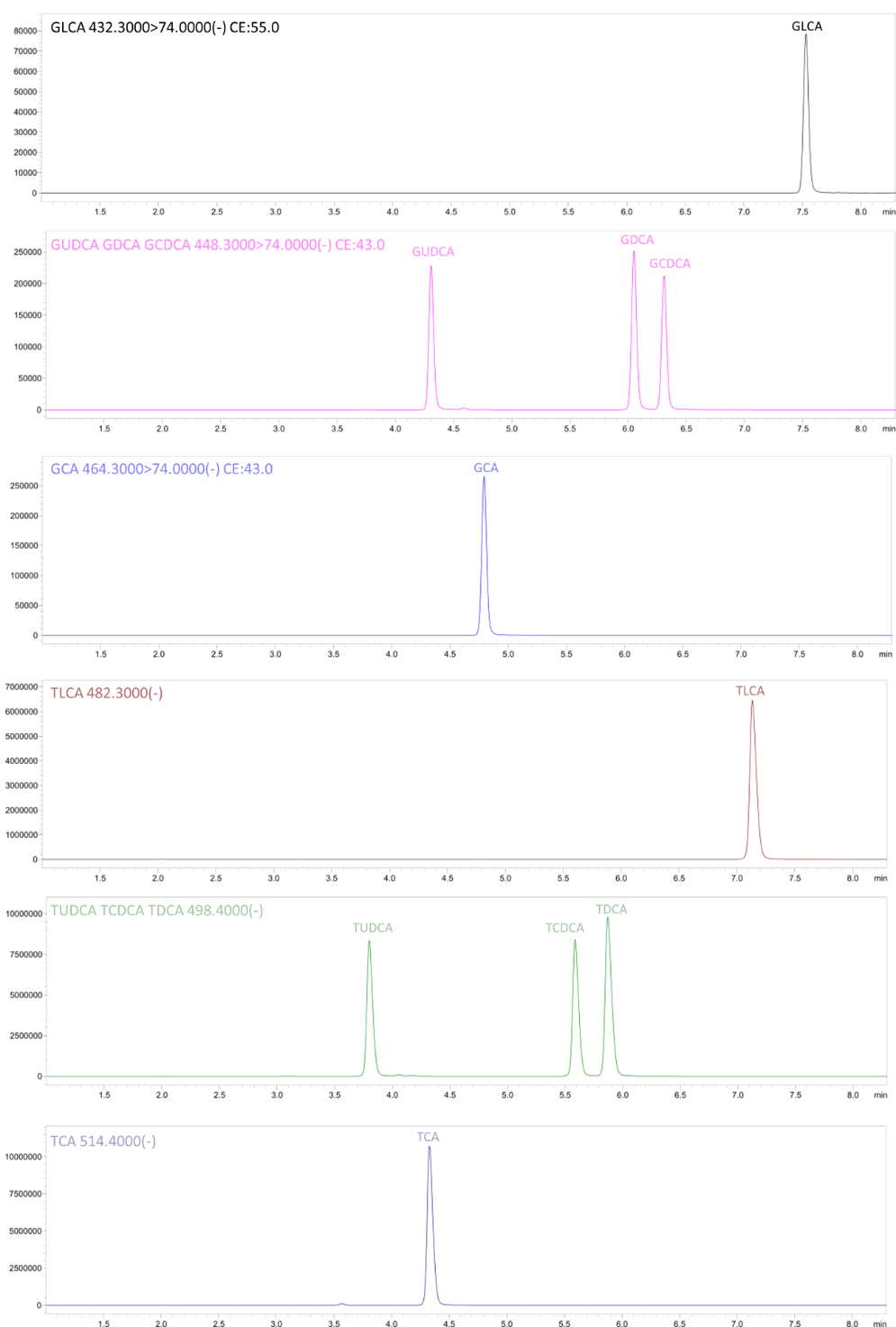


**Fig. S1.** Chromatogram of bile acid profiling.

**Table S1** bile acid quantification

|  | Mode | Q1 | Q3 | Retention Time |
| --- | --- | --- | --- | --- |
| GLCA | MRM | 432.3 | 74 | 7.534 |
| GUDCA | MRM | 448.3 | 74 | 4.314 |
| GDCA | MRM | 448.3 | 74 | 6.064 |
| GCDCA | MRM | 448.3 | 74 | 6.32 |
| GCA | MRM | 464.3 | 74 | 4.792 |
| TLCA | SIM |  | 482.3 | 7.101 |
| TUDCA | SIM |  | 498.4 | 3.778 |
| TCDCA | SIM |  | 498.4 | 5.562 |
| TDCA | SIM |  | 498.4 | 5.853 |
| TCA | SIM |  | 514.4 | 4.302 |

**Fig. S2.** DON reduces the viability of the pre-confluent Caco-2 cells. The viability of Caco-2 cells following exposure to different concentrations of DON (0-10 µM, 48 h) was analysed by WST-1 assay.

**Fig. S3.** DON does not affect the expression of FXR in Caco-2 cell layers. The mRNA expression of FXR in Caco-2 cell layers following exposure to DMSO or 2 µM DON for 48 h was analyzed by RT-qPCR. Data were expressed as mean ± SD, n=3. *: significantly different from the control group (p < 0.05).


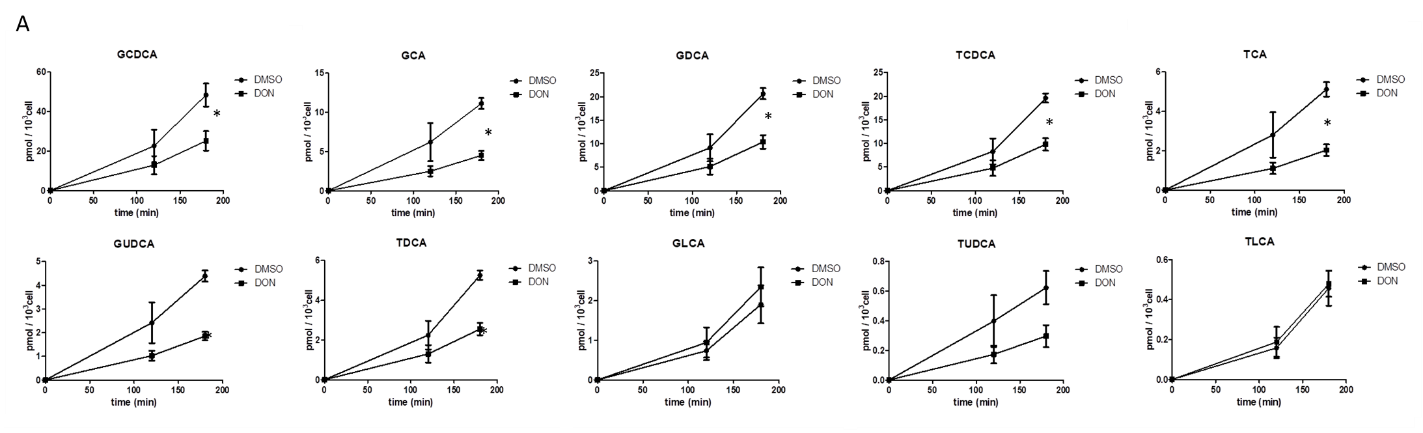


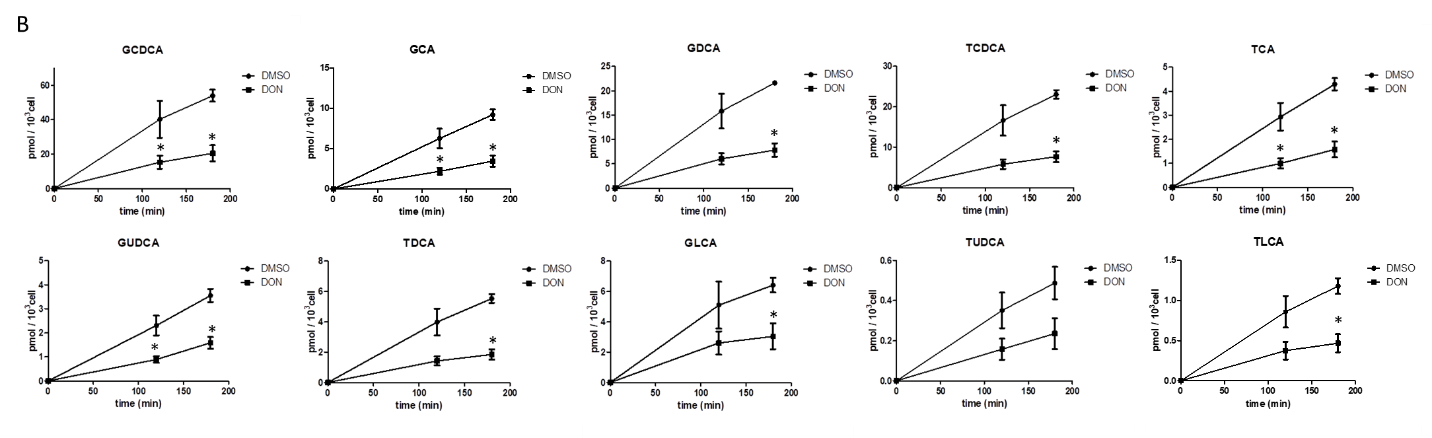


**Fig. S4.** DON decreases the conjugated bile acid transport through Caco-2 cell layers. Caco-2 cell layers were incubated with DMSO or DON (2 µM, 48 h) before exposure to different concentration of physiological mix of conjugated bile acids. (A) The time course of each conjugated bile acid transport following 250 nmol mixed conjugated bile acids. (B) The time course of each conjugated bile acid transport following 500 nmol mixed conjugated bile acids. Data were expressed as mean ± SD, n=3. *: Mean values differ significantly between the control group at same time point (p < 0.05).
